# Super-Resolution Imaging Reveals the Nanoscale Distributions of Dystroglycan and Integrin Itga7 in Zebrafish Muscle Fibers

**DOI:** 10.3390/biomedicines11071941

**Published:** 2023-07-08

**Authors:** Komala Shivanna, Mary Astumian, Prakash Raut, Vinh-Nhan Ngo, Samuel T. Hess, Clarissa Henry

**Affiliations:** 1 Department of Physics & Astronomy, University of Maine, 5709 Bennett Hall, Orono, ME 04469-5709, USA; komala.shivanna@maine.edu (K.S.); prakash.raut@maine.edu (P.R.); vinhnhan.ngo@maine.edu (V.-N.N.); 2 School of Biology and Ecology, University of Maine, 217 Hitchner Hall, Orono, ME 04469-5751, USA; mary.astumian@maine.edu

**Keywords:** super-resolution, FPALM, zebrafish, muscle fibers, localization microscopy, muscular dystrophy, fluorescence, *Danio rerio*, Dystroglycan, integrins

## Abstract

Cell signaling is determined partially by the localization and abundance of proteins. Dystroglycan and integrin are both transmembrane receptors that connect the cytoskeleton inside muscle cells to the extracellular matrix outside muscle cells, maintaining proper adhesion and function of muscle. The position and abundance of Dystroglycan relative to integrins is thought to be important for muscle adhesion and function. The subcellular localization and quantification of these receptor proteins can be determined at the nanometer scale by FPALM super-resolution microscopy. We used FPALM to determine localizations of Dystroglycan and integrin proteins in muscle fibers of intact zebrafish (*Danio rerio*). Results were consistent with confocal imaging data, but illuminate further details at the nanoscale and show the feasibility of using FPALM to quantify interactions of two proteins in a whole organism.

## 1. Introduction

Skeletal muscle plays a significant role in quality of life. Skeletal muscle mass and quality impact strength, mobility, locomotion, and health. For example, grip strength is predictive of healthy aging [1] and is better at stratifying the risk of cardiovascular disease than blood pressure [2]. Thus, understanding the molecular mechanisms that underlie skeletal muscle development, homeostasis, and aging is an high impact area of investigation. Skeletal muscle cells adhere to their surrounding extracellular matrix (ECM) as well as the ECM at the myotendinous junction [3,4]. Adhesion of muscle fibers to the ECM is critical for skeletal muscle function [4,5]. Genetic disruption of muscle fiber-ECM adhesion underlies multiple congenital muscular dystrophies (CMDs) [6,7,8,9] which are debilitating muscle wasting diseases with no cure.

Clearly, adhesion of muscle cells to their ECM microenvironment is critical for muscle development and homeostasis. Cell–ECM adhesion complexes are hubs that integrate chemical and mechanical information to regulate physiology. The two major transmembrane receptors that anchor mature muscle cells to their ECM microenvironment are Dystroglycan (DG) and integrin alpha7 (Itga7). Mutations in each of these receptors lead to congenital muscular dystrophies [10]. Normally, Itga7 dimerizes with integrin beta1 and binds to laminin in the basal lamina surrounding the skeletal muscle fibers. Laminin-211/221 is a heterotrimeric extracellular matrix protein essential for muscle homeostasis [11,12]. Dystroglycan is known to interact with laminin-211/221 [13]. Itga7 and DG display some degree of functional redundancy in both zebrafish and mouse models [14,15,16,17]. One therapeutic strategy involves promoting complementary, alternative mechanisms of cell–ECM adhesion. For example, addition of NAD+ to zebrafish deficient in either DG or Itga7 reduces dystrophy but has no impact on zebrafish deficient for both DG and Itga7 [14]. These data led us to hypothesize that NAD+ promotes increased adhesion of one receptor to laminin in the absence of another receptor by increasing clustering/activation of the other receptor. 

One hurdle to understanding the interactions of Itga7 and DG is that the nanoscale organization of these proteins in the vertebrate skeletal muscle membrane (sarcolemma) is not well understood. Specifically, it is crucial to determine the nanoscale organization of DG in the sarcolemma in intact zebrafish skeletal muscle. It is known that DG concentrates at the myotendinous junction (MTJ), which is the major site of force transmission from muscle to the skeletal system in rats, mice [18] and zebrafish model systems [19]. Failed adhesion of muscle fibers to their MTJ is a defining pathology in certain zebrafish models of muscular dystrophies [20,21]. DG also concentrates at costameres [18,22], subsarcolemmal structures at the intersection of t-tubules and sarcomeres as seen in zebrafish [23] mice [24], rat [18] and rabbit [25] models. Super-resolution stimulated emission depletion (STED) microscopy indicates that DG colocalizes with the insulin receptor at costameres [24], but other membrane domains were not examined. Thus, the nanoscale organization of DG in the zebrafish sarcolemma is not fully elucidated. In addition, it is not known whether the Dystroglycan complex (DGC) colocalizes with the integrin alpha7 complex, or if there are discrete sarcolemmal subdomains occupied by single complexes. Although DG and Itga7 interact via sarcospan as assessed by succinylated wheat germ agglutinin (sWGA) lectin chromatography, it is not known if these two adhesion complexes colocalize in vivo [26]. Given that both DG and Itga7 are critical for normal muscle development and homeostasis and can partially compensate for each other, it is imperative to elucidate the nanoscale organization of these receptors in wild-type muscle to provide a baseline for understanding how sarcolemmal organization is altered in disease states. 

Conventional antibody staining and visualization with confocal microscopy cannot determine the nanoscale organization of DG or whether DG and Itga7 interact because of the nanometer length scales involved; conventional light microscopy is limited by the diffraction of light to a resolution of ~200 nm, which is comparable to or larger than many relevant subcellular structures, rendering them too small to be observed in detail. For this reason, the nanoscale spatial distributions of several proteins involved in muscular dystrophy and other disease models remain largely unknown. The diffraction limit is overcome by super-resolution techniques including fluorescence photoactivation localization microscopy [27] (FPALM), photoactivated localization microscopy [28] (PALM) and stochastic optical reconstruction microscopy [29] (STORM), point accumulation for imaging in nanoscale topography [30] (PAINT) and DNA-based point accumulation for imaging in nanoscale topography [31] (DNA-PAINT). These techniques and others achieve resolutions ranging from 5 to 50 nm. Recently, minimal photon flux (MINFLUX) combines STED and single molecule localization microscopy (SMLM) to achieve further improvements in resolution [32]. 

Here, we leverage FPALM to determine, for the first time, the nanoscale organization of DG in skeletal muscle in an intact vertebrate, the zebrafish embryo. FPALM improves the resolution of optical microscopy, allowing for spatial resolution of a few tens of nm or better [33,34]. While it is known that DG concentrates at costameres, the sarcolemma, and at the MTJ, the relative density and size of DG clusters at these locations has not been elucidated. Here, we investigate the density, size, and organization of DG clusters in intact skeletal muscle. We hypothesized that DG clusters would be the most dense and largest at the MTJ, as this is the major site of force transmission from muscle to the skeletal system. Indeed, this was the case. Interestingly, the next most dense location of Dystroglycan was not at costameres, but at the sarcolemma. Finally, we show for the first time that Itga7 and DG colocalize at the single molecule level in intact skeletal muscle. Taken together, these data show the first in vivo nanoscale organization of DG and Itga7 in zebrafish. These results provide the basis for future assessment of the nanoscale organization of DG in the context of muscle diseases.

## 2. Materials and Methods

### 2.1. Sample Preparation

#### 2.1.1. Transposase and Clone Generation

Transposase mRNA was made by in vitro transcription using SP6 polymerase (mMESSAGE mMACHINE Kit, Invitrogen, Waltham, MA, USA) with Not1 linearized pCS2-TP plasmid kindly provided by Koichi Kawakami [35]. The resulting RNA was purified with the RNeasy Mini Kit (Qiagen, Germantown, MD, USA). The pME-Dystroglycan entry clone was created by designing gated primers: 5′- GGGGACAAGTTTGTACAAAAAAGCAGGCTATGCGCAATAAACTCA-3′ F primer and 5′-GGGGACCACTTTGTACAAGAAAGCTGGGTCGGGTGGCACGTAAG-3′ R primer. Zebrafish cDNA was used as the template in the gated PCR, followed by BP clonase II recombination with pDONR221 vector (Invitrogen, Waltham, MA, USA), transformation of TOP10 competent cells, followed by plasmid prep with the Qiagen Mini Prep kit. The p3E-Dendra2 entry clone was created by using Dendra2-TOM20 plasmid [36] as a template for a gated PCR to obtain Dendra2. Primers used were: 5′-GGGGACAGCTTTCTTTGTACAAAGTGGCAATGAACACC-3′ (forward) and 5′-GGGGACAAACTTTGTATAATAAAGTTGTTACCACACCTGGCT-3′ (reverse). Recombination with pDONRP2R-P3 (Invitrogen, Waltham, MA, USA created this entry clone. The *myogenin:integrin alpha 7: PAmKate* fusion clone was created similarly but used a PAmKate-containing plasmid as the template for a PCR with the following PAmKate primers: 5′-GGGGACAGCTTTCTTTGTACAAAGTGGTCATGTCTGAGCTGAT-3′ (forward) and 5′-GGGGACAACTTTGTATAATAAAGTTGCTAGTTCAGTTTGTGCCCCAGTTTGCT-3′ (reverse). All primers were purchased from Integrated DNA Technology (Coralville, IA, USA). The myogenin plasmid was a kind gift from Myron S Ignatius (UT Health, San Antonio, TX, USA). The fusion expression clone *beta-actin:Dystroglycan-Dendra2* was generated via Tol2 gateway cloning and LR clonase II and combined p5E-beta-actin from the Tol2Kit (kindly provided by Kawakami lab, Mishima, Japan), pME-dystrolgycan entry clone, p3E-Dendra2 entry clone, and pDestTol2pA2 destination clone from the Tol2Kit. The *myogenin:human integrin alpha 7-PAmKate* clone was similarly generated. The resulting clones were validated with Sanger sequencing. 

#### 2.1.2. Zebrafish Husbandry and Injections

Zebrafish were grown at 28.5 °C on a 14-h light/10-h dark cycle. Zebrafish embryos were collected from mass spawnings and raised in 1× Embryo Rearing Media supplemented with methylene blue to prevent microbe growth. Wildtype and *casper* embryos at the one-cell stage were co-injected with 1–2 nl of a mixture of 30 ng/μL final concentration *Tg:beta-actin:Dystroglycan-Dendra2* DNA or, for two-color, *Tg:myogenin:Dystroglycan-Dendra2/Tg:myogenin:integrin:PAmkate,* and 30 ng/ul final concentration of transposase RNA (transcribed from DNA kindly provided by Kawakami lab, Mishima, Japan) [35]. We found that zebrafish injected with the *beta-actin* promoter construct rather than the *myogenin* promoter construct, made more fibers that expressed Dystroglycan:Dendra2 and continued to use the *beta-actin* promoter construct in the following injections. After injection, all procedures were performed in the dark and embryos were grown in the dark to avoid photoconverting the Dendra2 protein. Embryos were screened at 24 h post-fertilization (hpf) for Dendra2 expression. At the appropriate time point of 48- or 72-h post-fertilization (hpf), Dendra2 positive embryos were manually dechorionated, fixed in 4% paraformaldehyde at room temperature for 2–4 h, and rinsed 5 × 5′ in 1× phosphate buffered saline (PBS). 

Background fluorescence caused by the natural pigmentation of the zebrafish epithelium was reduced by suppressing pigmentation. This was accomplished in wildtype embryos by phenylthiourea (PTU) treatment before 24 h post-fertilization which prevented some pigmentation. Pigmentation was already reduced in *casper* mutants, which are mutant for *nacre* and *roy* genes, and so lack all melanophores and all iridophores [37]. Although *casper* still contain xanthophores, they required no PTU treatment as their background fluorescence was already reduced enough to enable single molecule fluorescence microscopy. All protocols were performed following the University of Maine Institutional Animal Care and Usage Committee’s guidelines (IACUC number A3754-01 D16-00448).

### 2.2. Localization-Based Super-Resolution Microscopy 

#### Fluorescence Photoactivation Localization Microscopy (FPALM)

**Figure 1:**
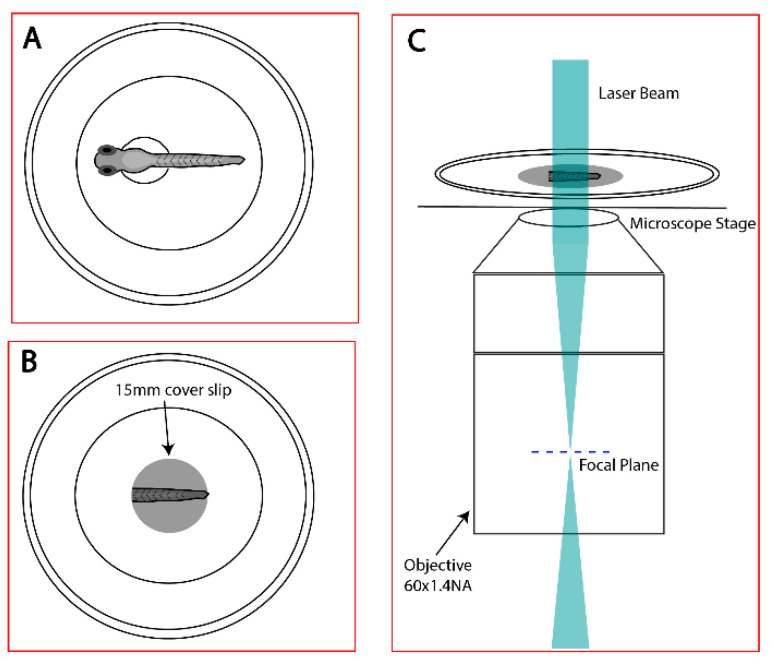
Schematic representation of the sample preparation right before FPALM imaging. (**A**) Fixed zebrafish in 35 mm MatTek dishes. (**B**) Deyolked zebrafish in same dish with 15 mm circular coverslip on top of the fish. (**C**) Sample placed on to the microscope stage with oil objective of 60× 1.4 NA.

**Figure 2:**
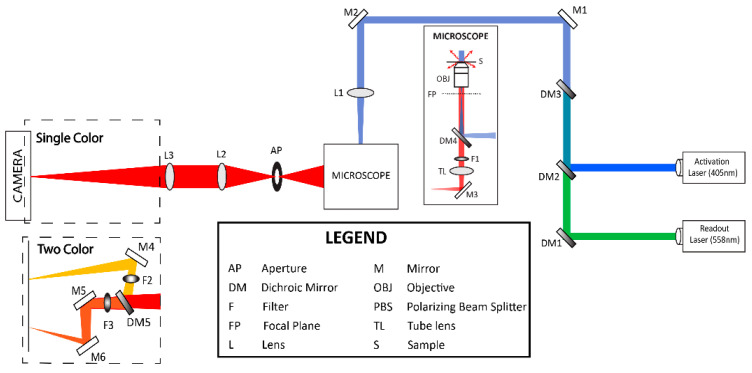
Experimental setup for single and two color FPALM in wide field illumination.

Fixed zebrafish embryos at 48–72 h post fertilization (hpf) were deyolked, to minimize background fluorescence due to yolk cells, and decapitated, to allow ease of mounting. The embryos were mounted during the imaging session, by transferring them to 35 mm dishes (MatTek, Ashland, MA, USA) in 1× PBS. A 15 mm circular coverslip was placed on the embryos to hold them close to the bottom glass of the dish (Figure 1B). To minimize drift of the sample, any excessive PBS was removed, and embryos were centered by holding a kimwipe to the appropriate edge of the coverslip. The sample dish was then mounted on the FPALM setup (Figure 1C) [33]. Briefly, the experimental setup (Figure 2) consists of an Olympus (Tokyo, Japan) IX71 inverted microscope with a 60× 1.45 NA objective lens, 2× telescope in the detection path, and an electron multiplying CCD (iXon+ DU897-DCS-BV, Andor/Oxford Instruments, Belfast, Ireland). The illumination path for FPALM consists of an activation laser with λ = 405 nm and excitation laser with λ = 561 nm aligned co-linearly. For better activation and readout, both lasers pass through a quarter wave plate (10EP54-1B, Newport, Irvine, CA, USA) to produce approximately circularly polarized light at the sample [38], followed by a convex lens of focal length 350 mm located at one focal length from the objective back aperture plane, which produced approximately widefield illumination at the sample. A quad band dichroic (Di01 R405/488/561/635, Semrock/IDEX, Rochester, NY, USA) in the turret of the microscope reflects the laser light into the objective. Fluorescence emission collected by the objective was filtered through the same dichroic and also a notch filter (Stopline 405 nm and 561 nm, Semrock/IDEX, Rochester, NY, USA), passed through the tube lens, followed by a 2× telescope, and a 585/40 emission filter (FF01-585/40-25, Semrock/IDEX, Rochester, NY, USA), before reaching the camera. For imaging of multiple fluorescent species, we followed previously published methods [33] whereby emitted light is passed through the same optical path up to but not including the emission filter, which is replaced with a multicolor module containing a dichroic mirror (FF580-FDi02-t3, Semrock/IDEX, Rochester, NY, USA), which splits the fluorescence into “green” (reflected) and “red” (transmitted) channels. Each channel then passes through an emission filter (red channel: ET605/70m, Chroma Technology Corp., Bellows Falls, VT, USA); Green channel: FF01-585/40-25, Semrock/IDEX, Rochester, NY, USA). Each channel then forms a separate image on the EMCCD.

### 2.3. Acquisition Parameters

The intensities of both readout and activation lasers are matched to the properties of the photoactivatable probe, considering their absorption coefficients and quantum yields for activation [39]. For the imaging of muscle fibers, the laser power (~30 mW for the 561 nm readout laser and ~100 μW for the 405 nm activation laser, measured at the objective) was varied to enable visibility of single molecules and minimize background (<80 photons per pixel per frame). To optimize the number of detected photons per frame and minimize the number of background photons collected, camera exposure time was adjusted in such a way that a single molecule was visible for approximately one frame before the bleaching of the fluorophore. Images were acquired with a frame rate of 40–50 Hz and electron multiplying gain of 200. 

### 2.4. Identification of Regions of Interest 

To the FPALM setup, an additional path was added for the blue LED lamp. The LED lamp system consists of a high-power blue LED (Odlamp: Blue 450–460 nm LED 50 W), heat sink (aluminum heat sink 3.15 × 3.15 × 1 inch), and voltage source (Naweisz: 60 V 5 A DC power supply). To illuminate the sample, the blue LED (emission peak 450–460 nm) light was passed through a 450–480 nm bandpass filter and then onto the turret dichroic. The sample was placed on the microscope stage with the sample region of interest roughly centered on the objective. Then muscle fibers were screened for the expression of the green fluorescence of the labeled protein of interest, in this case Dystroglycan-Dendra2. Regions to image were carefully chosen to avoid background fluorescence. Muscle fibers glowing faint to moderate green under the conditions described above represented sufficient transgenic expression of the plasmid and were considered optimal for imaging. In selecting the ROI to image, regions were omitted if two or more muscle fibers overlapped, since in this case the out-of-focal plane fiber created too much background fluorescence for the in-focal plane fiber to be imaged well. Additionally, if fibers had large glowing fluorescent puncta/blebs, they were rejected for similar reasons. High background levels in wildtype (pigmented) zebrafish precluded their use, necessitating the use of pigmentless *casper* mutants [40]. Most of the individual transfected fibers were found near the head of the fish. Muscle tissue towards the tail of the fish had relatively low numbers of fibers expressing the fluorescently labeled protein of interest. 

### 2.5. Identification and Localization of Single Molecules 

After the acquisition of data (image frames saved as tiff stack), raw images are then analyzed for molecular identification and localization using custom Matlab software version: 9.13.0 (R2022b) [41] with temporal median background subtraction [42] using a 100 frame window. Localizations were identified with a pixel intensity threshold of 20–50 photons [27], then sorted from highest to lowest intensities [43], then fitted using a two-dimensional Gaussian (Equation (11) from Hess et al. [27]) of the PSF to determine x position, y position, radius, amplitude, offset, and their respective errors. In the case of two-color data, threshold values are selected for each channel independently. After localization, the fit quality of each localization is screened for goodness of fit by requiring the values of the fitting parameters to be within certain ranges: Gaussian radius (1.0–3.2 pixels), localization precision (<100 nm), number of photons detected (200–3000), and fractional errors in the radius and Gaussian amplitude (both less than 30–50% error). Any localizations that fall outside the range are deemed to be a poor fit and are not included in further analysis. For such parameters, the average localization precision for the localizations is ~30 nm. To separate the different fluorescent labels in a multi-color sample, the α value (the ratio of transmitted photons over the sum of transmitted + reflected photons) is calculated for each localized PSF [33]. For this work, α values from 0 to 0.58 were assigned to Dendra2 and 0.59 to 1.0 to PamKate, respectively. 

### 2.6. Cluster Analysis

Cluster properties were extracted from rendered localizations [38]. To identify the clusters, molecules were rendered by creating a grid with 35 nm × 35 nm square pixels corresponding to the imaged region, and then counting the number of localizations within each grid pixel to create a density map. The density map is then convolved with a circle of radius of 50 nm normalized to unit area, which accounts for the localization error of the molecules. Subsequently, a mask for the imaged region of the muscle fiber was generated by automatic detection of the outer edge of the fiber using a previously published algorithm [34].The fiber area was then estimated using the summed area of the fiber mask, and the average localization density within the fiber was calculated from the number of molecules localized, divided by the fiber area. Clusters are determined by density thresholding at 1, 3, and 5 times above the average fiber density [34,44]. Cluster properties such as area, circularity and perimeter were extracted from masks of the cluster which were contiguous pixel regions above the applied density threshold using the regionprops function in Matlab.

## 3. Results

### 3.1. Dystroglycan in Skeletal Muscle Fibers

**Figure 3:**
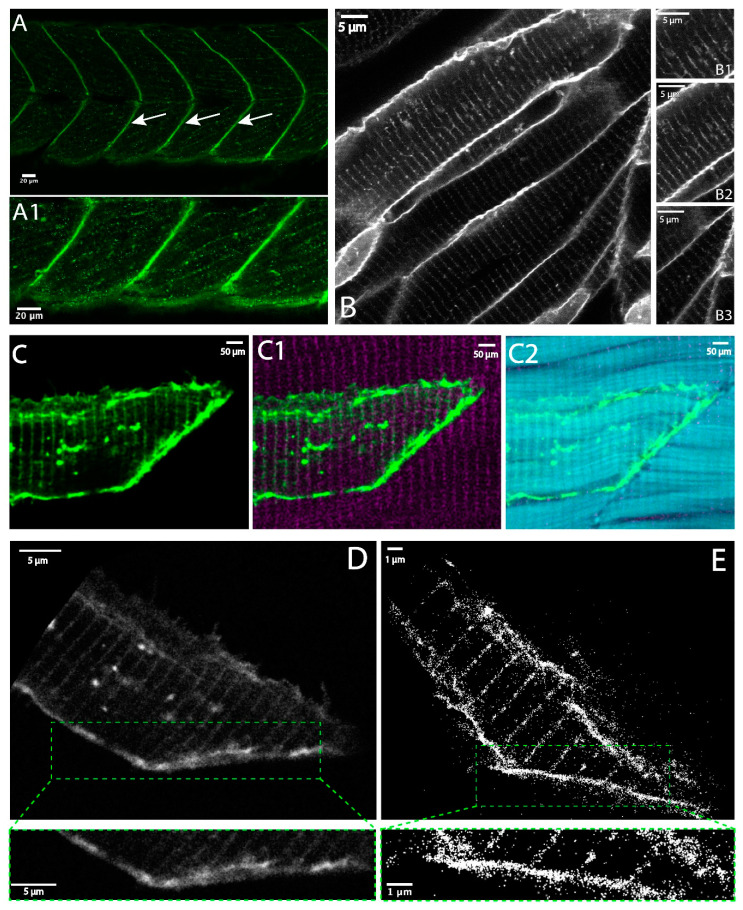
(**A**) Confocal image of a 72 hpf zebrafish antibody-stained for Dystroglycan, with Dystroglycan seen mainly at the myotendinous junction (MTJ), indicated by arrows. (**A1**) Confocal image shows Dystroglycan at both the MTJ and sarcolemma surrounding individual muscle fibers. (**B**) Confocal image shows transgenically expressed Dystroglycan–GFP is present at the (**B3**) MTJ, (**B2**) sarcolemma membranes, and (**B1**) t-tubules. (**C**) Confocal image of Dystroglycan-Dendra2 muscle fiber in green, (**C1**) merged Dystroglycan-Dendra2 muscle fiber in green with ryanodine receptor on t-tubules in magenta, and (**C2**) merged Dystroglycan-Dendra2 in green with phalloidin staining of actin in cyan (**D**) Confocal image of 48 hpf muscle fiber expressing Dystroglycan-Dendra2 fairly continuously at MTJ and t-tubule. (**E**) Super-resolution, FPALM image render, of 48 hpf muscle fiber expressing Dystroglycan-Dendra2, demonstrates that individual molecules can be localized and Dystroglycan can now be seen as clusters at the MTJ and t-tubule. Magnifications in the green boxed regions highlight the differing levels of information about the distribution of Dystroglycan at the MTJ that can be obtained from confocal and super-resolution images.

Immunohistochemistry staining for Dystroglycan at 72 hpf showed by confocal microscopy, that Dystroglycan concentrated at the MTJ (Figure 3A), and hints of Dystroglycan at the sarcolemma (muscle membrane) could be observed (Figure 3A1). The abundance of Dystroglycan at the MTJ made it difficult to visualize Dystroglycan at the sarcolemma or t-tubules with whole mount immunohistochemistry. Imaging Dystroglycan clusters in skeletal muscle fibers using localization microscopy (FPALM) required Dystroglycan tagged with a photoactivatable fluorescent protein such as Dendra2. Thus, we generated a construct, Tg:beta-actin:Dystroglycan:Dendra2 to allow single molecular visualization of Dystroglycan in intact skeletal muscle. To our knowledge, whether Dystroglycan overexpression could disrupt skeletal muscle development had not been determined. Therefore, we next asked: (1) whether overexpression of Dystroglycan:Dendra2 disrupted muscle development and homeostasis, and (2) if Dystroglycan:Dendra2 also localized to the MTJ, sarcolemma, and t-tubules. Overexpression of Dystroglycan:Dendra2 did not deleteriously impact zebrafish skeletal muscle development (Figure 3A1) as could be seen by normal somite boundary formation, phalloidin staining (Figure 3C2) and MTJ angles, averaging 103.8°, consistent with normal muscle development [14]. Confocal imaging of Tg:beta-actin:Dystroglycan:Dendra2 showed Dystroglycan at the MTJ (Figure 3B,B3,D), sarcolemma (Figure 3B,B2,D), and at striations, presumably t-tubules, within muscle fibers (Figure 3B–B3,D). To test whether Dystroglycan was indeed at the t-tubules, we stained Tg:beta-actin:Dystroglycan:Dendra2 injected embryos with a ryanodine receptor antibody (RyR1) that marks the t-tubules (Figure 3C1) [45]. Dystroglycan could be observed colocalizing with RyR1 (Figure 3C1), indicating that Dystroglycan was concentrating at t-tubules. We also visualized Dystroglycan at the muscle membrane by expressing Tg:mylz2:lyn:amcyan:Dystroglycan (Figure 3B–B3).These data indicated that Dystroglycan:Dendra2 expression did not impact skeletal muscle development and reflected the distribution of endogenous Dystroglycan. Confocal imaging of Dystroglycan:Dendra2 suggested that the Dystroglycan distribution at the MTJ, t-tubules, and sarcolemma was contiguous (Figure 3D). However, one would have predicted that there would be clusters of Dystroglycan mediating adhesion to the ECM. FPALM images showed discrete Dystroglycan clusters at the nanoscale in the different regions (t-tubule, sarcolemma, and MTJ) of muscle fibers (Figure 3E), demonstrating the additional information provided by enhanced resolution. 

### 3.2. Quantitative Comparison of Muscle Features Using Confocal and Super-Resolution Microscopy

**Figure 4:**
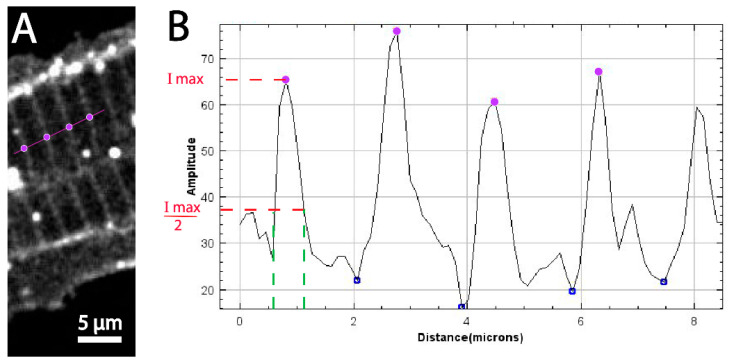
(**A**) Shows the cropped region of the muscle fiber imaged using the Leica DMi8 confocal and 25× water lens. Purple line with circles indicates the regions where the intensities of the pixels are measured. (**B**)Two-dimensional graph of intensities of pixels along a line within the image. X-axis represents distance in microns and Y-axis represents amplitude intensity. Purple dots show the position of maximum intensity and distance between maximum intensity represents the distance between the t-tubules. The width of the t-tubule (represented by green dotted line) was obtained by measuring the width at half maximum of the intensity peak, represented by a red dotted line.

**Figure 5:**
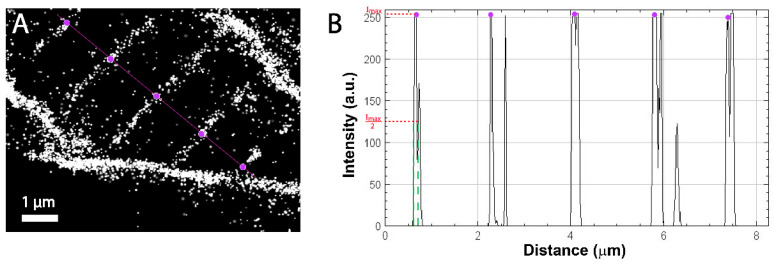
(**A**) Cropped region of the muscle fiber imaged using FPALM. The purple line shows the path along which the intensities of the pixels are measured. Purple circles show positions of intensity peaks. (**B**) Plot of pixel intensities vs. distance along the line shown in A. Purple dots show the positions of maximum intensity. The distance between intensity maxima represents the distance between the t-tubules. The apparent width of the t-tubule (represented by the green dotted line) was obtained by measuring the full width at half maximum of the intensity peak, represented by the red dotted line.

We observed distinct localizations and clusters of Dystroglycan at the t-tubules and MTJ (Figure 3E). We measured the average distance between the t-tubules making up the muscle cell membrane in both the confocal images and the super resolution images by using the measure tool and plot profile on ImageJ [46]. From the measuring tool, lines were drawn across the t-tubule and the distance between each t-tubule was measured. Plot profile created a plot of pixel values relative to background across the t-tubules on images as shown in Figure 4A and Figure 5A obtained from both confocal and FPALM imaging, respectively. Figure 4A and Figure 5A show the plot of pixel values along the line drawn across multiple t-tubules. Figure 4B and Figure 5B display a two-dimensional graph of the intensities of pixels along the muscle fiber within the FPALM and confocal image. The x-axis represents distance across the striated structure (purple line as shown in Figure 4A and Figure 5) and the y-axis represents pixel intensity. Measuring distance between the peaks gave the distance between the t-tubules as shown in Figure 4B and Figure 5B by the purple dots. We found the average distance between each t-tubule was 1.84 ± 0.09 μm from the confocal image (see Table 1) and about 1.722 ± 0.076 μm in the super- resolution image Table 2. Measuring the full width half maximum of the intensity peaks (Figure 3B and Figure 4B red dashed line) gave the approximate size of the t-tubules convolved with the resolution of the microscope being used. From the confocal image, the apparent width of the t-tubule was determined to be 380 ± 60 nm and from the FPALM image was determined to be 57 ± 1 nm, which includes both the actual width of the structure and the resolution of the given microscopy technique.

### 3.3. Thresholding by Density Indicates Dystroglycan Is Concentrated at the MTJ 

**Figure 6:**
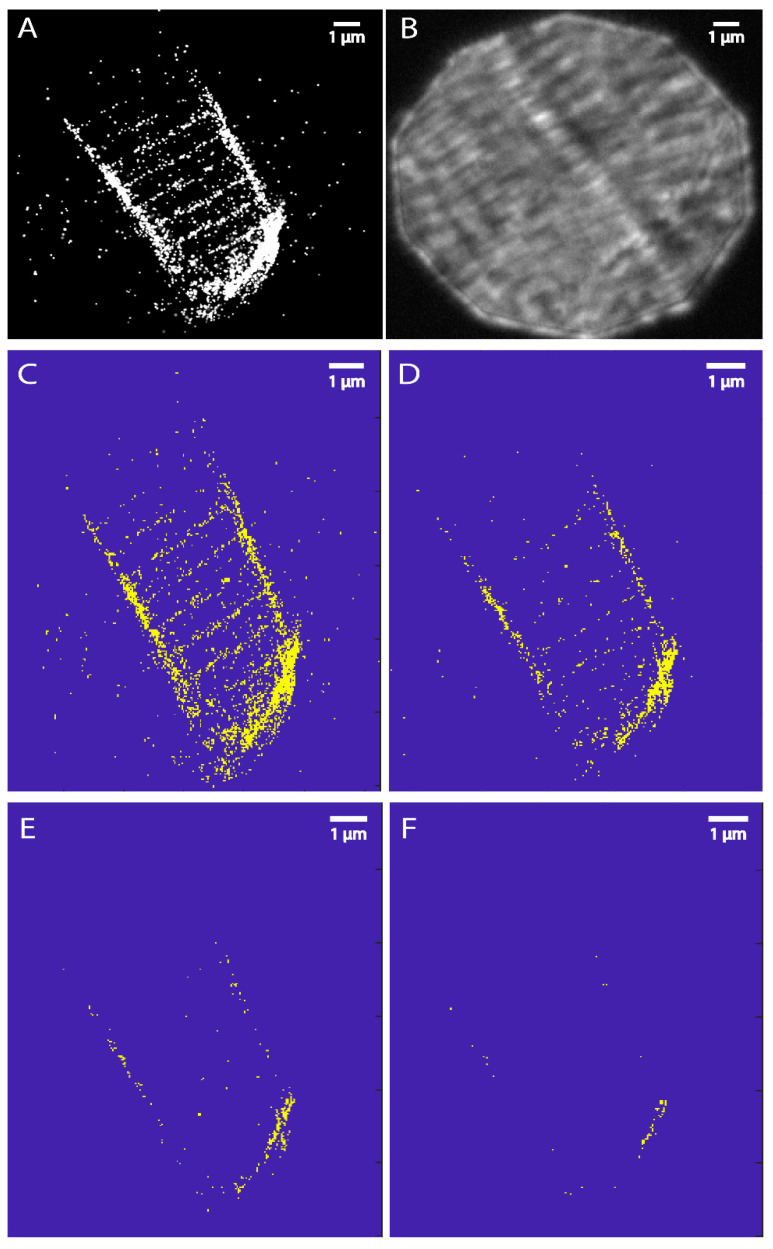
(**A**) FPALM image showing Dystroglycan-Dendra2 localizations in whole zebrafish muscle fiber and (**B**) its corresponding brightfield image. (**C**) Dystroglycan–Dendra2 localizations are seen in the MTJ, sarcolemma, and t-tubule regions without density thresholding. (**D**) Localizations thresholded to those areas above the average localization density (localizations per μm^2^). (**E**) Localizations after thresholding at or above three times the average density show few remaining at the t-tubule, indicating lower density in the t-tubule region. (**F**) The remaining localizations after thresholding at or above five times the average density indicate that the MTJ is the most dense region for localizations, followed by the sarcolemma.

**Figure 7:**
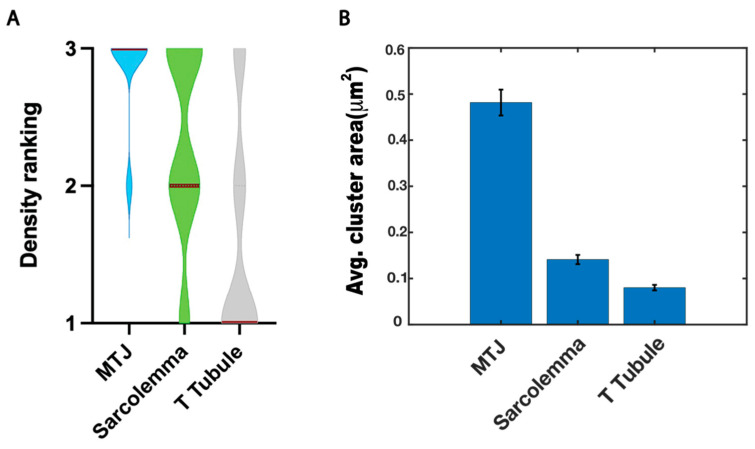
(**A**) Binning Dystroglycan densities by region on muscle fibers, with 3 as most dense and 1 as least dense. (**B**) Average cluster area of Dystroglycan clusters in MTJ, sarcolemma and t-tubule regions estimated by cluster analysis.

The above data clearly show that Dystroglycan:Dendra2 is clustered at three main locations in skeletal muscle fibers: the MTJ, sarcolemma, and t-tubules. The forces that impact these locations are different. For example, the MTJ is the major site of force transmission from muscle to the skeleton [47,48], and thus we would predict that cluster size and/or density would be highest at the MTJ. We used thresholding by density to elucidate the regions with the densest Dystroglycan. Thus, regions were thresholded based on local density (number of localizations/μm^2^) at or above 1×, 3×, or 5× the average density. Examples of these thresholds are shown in Figure 6. These data suggested that, on average, density at the MTJ was higher than at the sarcolemma and t-tubules (as indicated by the majority of localizations at 5× the average density being at the MTJ in Figure 6F). The thresholded images were used to qualitatively score the comparative Dystroglycan:Dendra2 density at the three major locations: the MTJ, sarcolemma, and t-tubules. The region with the most dense threshold was scored 3, the next most dense region was scored 2, and the least dense region was scored 1. In Figure 6 panel F it is clear that the most dense region was the MTJ. Panel E shows thresholding at 3× the average density. At this threshold, more localizations were observed at the MTJ than in panel F (5× the average density). In addition, localizations were observed at the sarcolemma at this threshold. Panel D shows thresholding with density at or above the average density whereby localizations were observed at t-tubules in addition to the MTJ and sarcolemma. This process was repeated for 15 fibers and results are shown in Figure 7A. Taken together, these data showed that Dystroglycan clusters are most dense at the MTJ, the clusters are of medium density at the sarcolemma, and Dystroglycan clusters are least dense in the t-tubules. These structural details and individual localizations are inaccessible to confocal imaging. 

The above data show the density distribution of Dystroglycan:Dendra2 in skeletal muscle. We next asked whether discrete clusters of Dystroglycan:Dendra2 were larger at the MTJ by conducting cluster analysis of the FPALM renders. To estimate the cluster areas of Dystroglycan:Dendra2 at the MTJ, Sarcolemma and t-tubules we considered regions that were above 3× the average density. Figure 7B shows the average cluster area at MTJ was 0.481 ± 0.028 μm^2^, sarcolemma was 0.141 ± 0.01 μm^2^ and t-tubules was 0.080 ± 0.006 μm^2^_,_ respectively. From the cluster area estimation we found that the Dystroglycan:Dendra2 clusters were larger at the MTJ which was also shown by the density maps (Figure 6F). From our results we estimated the localization precision σ_xy_ = 44 nm and nearest neighbor distance r_NN_ = 22 nm, allowing us to estimate that the approximate resolution of the FPALM images was 49 nm, according to $R\sim \sqrt{\sigma_{\mathrm{xy}}^{2}+r_{\mathrm{NN}}^{2}}$ [39]. 

### 3.4. Super-Resolution Microscopy of the Co-Distribution of DG and Itga7 

**Figure 8:**
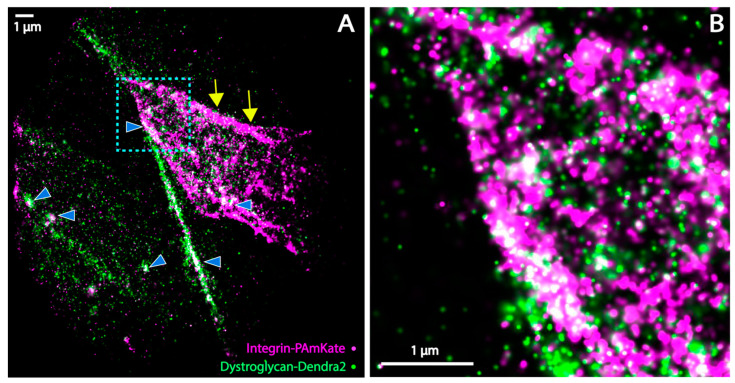
(**A**) Two-color FPALM image rendered from muscle fiber expressing Dystroglycan-Dendra2 and Itga7-PAmKate. Areas of colocalization of Dystroglycan and integrin are shown by blue filled carats. Clusters of Itga7 alone are shown by yellow arrowheads. (**B**) Magnified view of the region in the cyan box from panel A highlights colocalizations of Dystroglycan and integrin at the MTJ. Scale bar = 1 μm.

One major gap in our understanding of skeletal muscle structure was whether DG and Itga7 interact or colocalize at the nanoscale level. Colocalization of DG and Itga7 was possible because both proteins bound to overlapping and exclusive domains of laminin [49] present in the ECM surrounding muscle fibers. In order to answer these questions, we generated a construct encoding *itga7:PAmKate* in order to have spectrally separated photoactivatable fluorescent proteins for both DG and Itga7. This enabled simultaneous imaging of DG and Itga7 proteins as seen in Figure 8. Colocalization of DG and Itga7 in the muscle fiber and MTJ was seen as white regions in Figure 8 with areas of dense colocalization marked by blue carats. We observed strong colocalization between DG and Itga7 near where the muscle fiber attaches to the MTJ and occasionally in the sarcolemma (Figure 8). Using data from three independently imaged regions, we found 25.3 zones of colocalization per region with an estimated size of 80–120 nm.

## 4. Discussion

Muscle fiber adhesion is important for overall health. Muscle adhesion is mediated by the proteins Dystroglycan and integrin, which connect the muscle fiber to the surrounding ECM. Dystroglycan was known to localize to the zebrafish MTJ, sarcolemma, and t-tubule by confocal imaging and electron microscopy, but the exact distribution of the Dystroglycan at these structures in zebrafish muscle was unknown. Our FPALM imaging and analysis shows that Dystroglycan localizes in clusters with the most dense clusters at the MTJ, medium density at the sarcolemma surrounding the muscle fiber, and lower density at the t-tubule (Figure 6 and Figure 7). The ability to distinguish Dystroglycan clusters of various densities illustrates the advantages of super resolution compared to confocal for understanding nanoscale structure and organization of the muscle membrane. Electron microscopy enables finer resolution and has been used to image zebrafish muscle, but samples must be fixed and thinly sectioned first, meaning that the intact zebrafish embryo’s muscle cannot be imaged [50,51,52]. In addition to the cluster density of Dystroglycan at various points in the muscle fiber, we investigated whether colocalization of Dystroglycan and integrin occurred. Colocalization was expected due to molecular evidence of the interaction of Dystroglycan and integrin proteins and due to their mutual binding to laminin in the ECM, but it was not known previously if Dystroglycan and integrin colocalized in muscle. We found by FPALM that there is colocalization of DG and Itga7 at both the MTJ and in the muscle fiber (Figure 8). 

### Impact of Super Resolution on Elucidating Nanoscale Structure in the Zebrafish Model 

Super resolution imaging opens the possibility for nanoscale imaging of proteins, protein–protein interactions, and other molecules of interest in a living model organism. Super resolution is increasingly used in intact zebrafish, which are an excellent model system to study muscle development, structure, function, and related human muscle diseases [53,54]. Other examples of super resolution imaging used in zebrafish include the use of STED to visualize the ribbon synapse protein in retinas [55]; nonlinear structured illumination microscopy combined with two-photon Bessel light-sheet to image podocytes, specialized kidney epithelial cells [56,57]; point accumulation for imaging of nanoscale topography (PAINT) to image neuromast and surrounding striated muscle fibers [58]; and the use of FPALM to probe mechanisms of influenza viral infection [44]. Adding to the growing literature of super resolution findings in zebrafish, here our use of FPALM provides new insight into the biological organization of transmembrane proteins. Other super-resolution methods could have been used also to visualize the same structures, but each super-resolution technique has distinct strengths and limitations. During the stage of experimental design, it is vital to consider the temporal and spatial resolution needed to inform the choice of a particular super resolution technique to best answer each biological question. Rather than picking the most resolved technique, the required resolution is determined by considering the size of the object, the wavelength and acquisition time which impact the error of measurement, and the localization uncertainty which is affected by factors such as fluorophore photophysics, detection efficiency, and fluorescence background [59]. In our case, FPALM, a type of single molecule localization microscopy, is well-suited for imaging the length scale of tens of nanometers required to quantify the relative localizations of DG and Itga7 in the zebrafish muscle membrane. Simultaneous localization imaging of more than one protein species in the zebrafish embryo is an advance for super resolution microscopy. Following previous work by K. Gabor et al. with a single fluorescent species imaged *in vivo* [40], it will be exciting to attempt multicolor methods in living embryos.

Previously it was speculated that Itga7 and DG interacted, but there was no direct evidence of colocalization. Our FPALM imaging shows for the first time that Itga7 and DG colocalize at the muscle fiber and MTJ in zebrafish. Additionally, our cluster density analysis shows that Dystroglycan is present in clusters of varying average densities depending on the part of the muscle structure. While we found spatial variation in the Dystroglycan density and its highest levels at the MTJ, the next step will be to perform imaging in live zebrafish to visualize initial cluster formation and then cluster maturation and turnover. A mathematical model for initial integrin cluster formation predicts that integrin will form smaller clusters when ECM proteins are concentrated rather than spread out [60], but this model ignores growth, adhesion, maturation, and turnover. The turnover or mobility of the molecules could be assessed by using single molecule trajectory analysis [61] or fluorescence recovery after photobleaching FRAP [62]. Live imaging would allow observation of growth and maturation. The initiation and changes to the colocalization of DG and ITGa7 during muscle development and disease could also be tracked in living zebrafish. We are interested in human Dystroglycanopathies which are muscle diseases caused by aberrant glycosylation of the Dystroglycan protein. In Dystroglycanopathies, human patients have varying degrees of muscle disease symptoms. Severity of symptoms might be related to the relative DG disorganization in their skeletal muscle. The mechanisms and impacts of DG disorganization at the membrane are not well understood. It is possible that DG disorganization could also impact the localization of integrins, thus reducing any compensatory mechanisms for the muscle fiber to bind laminin. In the Dystroglycanopathy zebrafish model, in vivo FPALM would make it possible to test for a correlation over time between protein glycosylation, protein position in the muscle membrane, and later muscle fiber detachments which would be valuable for understanding this disease. Furthermore, these findings demonstrate the in-principle capability of imaging multiple biomolecules as a function of time in an intact organism for a variety of applications relating to disease.

## Figures and Tables

**Table 1:** Distance Between Adjacent t-Tubules Obtained by Confocal Microscopy.

Peak Position (μm)	Distance between Adjacent Maxima (μm)
0.84 ± 0.29	1.88 ± 0.29
2.72 ± 0.29	1.73 ± 0.29
4.46 ± 0.29	1.87 ± 0.29
6.33 ± 0.29	

**Table 2:** Distances Between Adjacent t-Tubules Obtained by Super-Resolution Microscopy (FPALM).

Peak Position (μm)	Distance between Adjacent Maxima (μm)
0.5914 ± 0.07	1.535 ± 0.07
2.127 ± 0.07	1.913 ± 0.07
4.040 ± 0.07	1.760 ± 0.07
5.800 ± 0.07	1.624 ± 0.07
7.442 ± 0.07	

## Data Availability

Raw image files supporting this study will be provided, upon reasonable request to one of the corresponding authors.
